# An interpretable measure of semantic similarity for predicting eye movements in reading

**DOI:** 10.3758/s13423-022-02240-8

**Published:** 2023-02-02

**Authors:** Sun Kun, Wang Qiuying, Lu Xiaofei

**Affiliations:** 1 Department of Linguistics, University of Tübingen, Tübingen, Germany; 2 School of Teaching, Learning and Educational Sciences, Oklahoma State University, Stillwater, United States; 3 Department of Applied Linguistics, The Pennsylvania State University, University Park, United States

**Keywords:** Contextual semantic similarity, Model comparison, Interpretability, Word predictions

## Abstract

Predictions about upcoming content play an important role during language comprehension and processing. Semantic similarity as a metric has been used to predict how words are processed in context in language comprehension and processing tasks. This study proposes a novel, dynamic approach for computing contextual semantic similarity, evaluates the extent to which the semantic similarity measures computed using this approach can predict fixation durations in reading tasks recorded in a corpus of eye-tracking data, and compares the performance of these measures to that of semantic similarity measures computed using the cosine and Euclidean methods. Our results reveal that the semantic similarity measures generated by our approach are significantly predictive of fixation durations on reading and outperform those generated by the two existing approaches. The findings of this study contribute to a better understanding of how humans process words in context and make predictions in language comprehension and processing. The effective and interpretable approach to computing contextual semantic similarity proposed in this study can also facilitate further explorations of other experimental data on language comprehension and processing.

**Supplementary Information:**

The online version contains supplementary material available at 10.3758/s13423-022-02240-8.

## Introduction

Language users are able to anticipate upcoming linguistic content. While prediction was traditionally considered to be an inefficient and cognitively expensive processing mechanism in language comprehension, an increasing body of evidence from empirical studies has gradually created a consensus in the research community that expectations or predictions about upcoming content play a very important role in language comprehension and processing (e.g., MacDonald, Pearlmutter, & Seidenberg, 1994; Kutas & Federmeier, 2011; Huettig, 2015; Kuperberg & Jaeger, 2016).

The evidence shows that comprehension can be facilitated when the upcoming words are highly predictable (DeLong, Urbach, & Kutas, 2005; Pickering & Garrod, 2007; Willems, Frank, Nijhof, Hagoort, & Van den Bosch, 2015). For example, people are faster at processing more predictable words (e.g., “picture books” in “the 3-year-old kid is reading *picture books*”) than less predictable ones (e.g., “physics books” in “the 3-year-old kid is reading *physics books*”) within the same grammatical context. Decades of research on human sentence processing have demonstrated that the time spent on a word in reading is usually indicative of the difficulty in processing the word, suggesting that reading time is a fair measure of reading difficulty. Reading time can be recorded using many devices, one of the most reliable and established of which is an eye-tracker (Liversedge, Paterson, & Pickering, 1998; Rayner, 1998; Rayner, Chace, Slattery, & Ashby, 2006; Schotter, Bicknell, Howard, Levy, & Rayner, 2014). The significance of the relationship between reading difficulty and eye movements has inspired a host of studies investigating the use of eye-tracking data with a view to improving and testing computational models of language (Barrett & Søgaard, 2015; Demberg & Keller, 2008; Klerke, Castilho, Barrett, & Søgaard, 2015).

The meanings of words can be represented using word vectors based on distributional semantics (Mitchell & Lapata, 2010), such as those yielded by word2vec programs or transformers (e.g., BERT) (Mikolov, Chen, Corrado, & Dean, 2013; Bojanowski, Grave, Joulin, & Mikolov, 2017; Devlin, Chang, Lee, & Toutanova, 2018). Recent studies have used different types of word vectors trained by various models to investigate reading behavior using datasets of eye-tracking, EEG and fMRI signals (Hollenstein, de la Torre, Langer, & Zhang, 2019; Hollenstein et al., 2021). Such studies have commonly employed *correlation*-based methods for model evaluation and comparison, which is inadequate for understanding the direct predictive effect of the word vectors on reading behavior in these datasets. A few other studies have examined the predictive effect of semantic similarity, a measure of the semantic connections or relations between words, sentences, documents, or concepts, on how words are processed, with the semantic similarity between words quantified as the distance between two word vectors (Rapp, 2002; Sahlgren, 2008); (Roland, Yun, Koenig, & Mauner, 2012). The use of this metric facilitates the employment of more effective statistical methods for assessing the direct effects of variables and comparing model performance. The semantic similarity of two words can be computed from their word vectors without taking context into account (Mitchell et al., 2008; Mitchell & Lapata, 2010; Westbury & Hollis, 2018). However, others have advocated for the use of contextual semantic similarity, which allows an individual word to obtain different values in different contexts, to better capture how words are processed in different contexts in experimental or naturalistic language comprehension settings. To differentiate from the semantic similarity of isolated words, we use the term *contextual semantic similarity* to refer to the semantic similarity between a target word and its neighboring words (e.g., the three preceding words) in naturalistic discourse in the current study. Although semantic similarity as a metric has had a wide range of applications in computational linguistics and artificial intelligence (see Harispe, Ranwez, Janaqi, & Montmain, 2015; Jabeen, Gao, & Andreae, 2020; Chandrasekaran & Mago, 2021), its use in cognition-related studies remains relatively limited.

Several influential approaches for computing contextual semantic similarity exist, such as the cosine (Frank & Willems, 2017) and Euclidean methods (Broderick, Anderson, & Lalor, 2019). As discussed in Section “Related work”, such approaches may be improved in terms of comprehensiveness in the types of neighboring words considered and/or the linguistic interpretability of the semantic similarity values obtained. The current study proposes a novel, dynamic approach for computing contextual semantic similarity aimed at addressing these issues. Our approach considers comprehensive linguistic contexts and computes the semantic similarity value following a set of steps that are mathematically and linguistically interpretable. We examine the effectiveness of the contextual semantic metric computed using this approach for predicting eye-tracking data from language comprehension tasks and compare the performance of this approach to that of the cosine and Euclidean approaches. More specifically, the present study is guided by the following two research questions:

1. Can the contextual semantic similarity metric computed using this new, dynamic approach predict eye-movements in reading English words?
2. How does the semantic similarity metric computed by the novel approach compare to those computed by cosine and Euclidean approaches in predicting eye-movements in naturalistic discourse reading?

## Related work

### Word vectors and semantic similarity in cognitive studies

Word vectors have been used to represent the meanings or contexts of target words. In earlier research, word vectors consist of dimensions corresponding to words in the vocabulary or documents in a collection. Recent research has increasingly employed word embeddings obtained through neural language models that are better at capturing the semantic and grammatical behaviors of words, such as those yielded by word2vec programs, which do not consider word order, and those obtained from pre-trained language models such as Embeddings from Language Models (ELMo) and Bidirectional Encoder Representations from Transformers (BERT), which consider word order (Mikolov et al., 2013; Bojanowski et al., 2017; Devlin et al., 2018). Many studies have reported that word vectors are closely related to neural and cognitive effects in language processing and reading comprehension. For instance, researchers have found neural correlates between the word vectors themselves (rather than the distances between them), in both single-word comprehension (Mitchell et al., 2008) and in narrative reading (Wehbe, Vaswani, Knight, & Mitchell, 2014). With the recent advancement of natural language processing (NLP) techniques and the availability of experimental databases on language comprehension, more studies have been done to test the feasibility of using word embeddings to predict reading behavior. For instance, (Abnar, Ahmed, Mijnheer, & Zuidema, 2018) evaluated the usefulness of eight word embedding models for predicting the neural activation patterns associated with concrete nouns. Hollenstein et al., (2019) evaluated how well six types of word embeddings could predict eye-tracking features, EEG, and fMRI signals using 15 datasets of eye-tracking, EEG and fMRI signals. Hollenstein et al. (2021) reported a shared task on eye-tracking prediction using word embeddings trained by different methods. These studies have contributed useful insight into the types of word embeddings that are useful for predicting reading behavior.

The studies discussed above have commonly employed *correlation*-based analysis to examine the relationship between word vectors or embeddings and reading behavior. While useful for measuring the relationship between two variables, the *correlation* analysis is weaker for testing the predictability effect of a variable or for comparing model performance than *regression* analysis (Bewick, Cheek, & Ball, 2003). In order to employ regression models to examine the predictability of word vectors for reading behavior, some researchers have converted word vectors to semantic similarity metrics (Frank & Willems, 2017; Broderick et al., 2019). The semantic similarity between word vectors has been shown to be predictive of semantic priming effects in word naming (Jones, Kintsch, & Mewhort, 2006) and in priming and lexical decision experiments (Günther, Dudschig, & Kaup, 2016; Lund & Burgess, 1996; Kumar, 2021). Additionally, (Pynte, New, & Kennedy, 2008) found that a larger LSA (Latent semantic analysis) distance between the current and previous content word(s) results in longer word-reading time on the French part of the Dundee corpus (Kennedy, Hill, & Pynte, 2003). LSA semantic similarity was also found to be useful in predicting language processing (Luke & Christianson, 2016; Roland et al., 2012). Notably, studies along this line have mostly examined isolated target words in such tasks as lexical naming, lexical decision, or lexical priming rather than real-world naturalistic discourse processing, in which the same word may be processed differently in different contexts.

In line with the observation that the semantic context can influence word processing (Ehrlich & Rayner, 1981; Hale, 2001; Smith & Levy, 2013), recent studies have increasingly used *contextual semantic similarity* measures computed from state-of-the-art word embeddings obtained through neural language models (Mikolov et al., 2013; Bojanowski et al., 2017; Devlin et al., 2018) to predict word processing in real-word naturalistic discourse processing tasks. Fixed semantic similarity measures computed for isolated words are inadequate for capturing the fact that the same words can be processed differently in different contexts in naturalistic discourse comprehension. Contextual semantic similarity measures are calculated for each instance of a target word in naturalistic discourse by taking into account of its contextual or neighboring words. We turn to a discussion of approaches to computing contextual semantic similarity in the next section.

### Contextual semantic similarity in language comprehension/processing

A number of approaches for computing semantic similarity have been proposed in the NLP literature, such as cosine, Manhattan distance, Kullback–Leibler divergence, Euclidean distance, and the product measure (Harispe et al., 2015; Lane, Howard, & Hapke, 2019; Chandrasekaran & Mago, 2021). Some of these approaches can be used to calculate the semantic similarity between isolated words, and the resulting measures have been shown to be predictive of semantic priming and lexical psychological properties (Mandera, Keuleers, & Brysbaert, 2017; Hollis & Westbury, 2016). Of interest to us here are two approaches for computing *contextual semantic similarity* that have been used to examine word predictions and processing in naturalistic discourse processing.

**Fig. 1:**
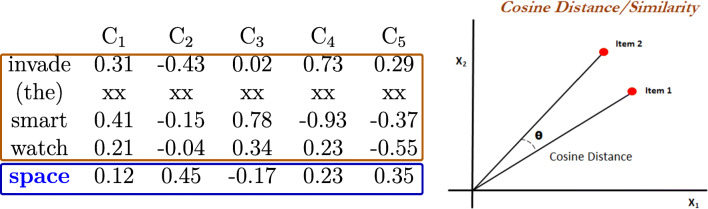
The cosine approach from Frank & Willems (2017). In the left table, the target word is “space”, and its preceding three content words are “invade”, “smart” and “watch” (the function word “the” is excluded). Each word has its own word vector, and the word embeddings of the three preceding content words are added up to yield a new vector, represented by “item 2” in the right panel. The vector of the target word “space” is represented by “item 1” in the right panel. The cosine distance between the two vectors is referred to as the *cosine semantic similarity* of the target word in the current study

The *cosine* approach is a simple but highly influential and practical measure of vector similarity (Jurafsky & Martin, 2008, 677–678; Lenci, 2018) that has been modified to assess semantic similarity in studies of word predictions and processing. Frank & Willems (2017) computed the contextual semantic similarity between a target word *w*^*t*^ and its preceding content words within the current sentence as *cos(A*, *w*^*t*^), where *A* denotes the sum of the vectors of its preceding content words.^1^ This cosine value is treated as the contextual semantic similarity value of the word *w*^*t*^, as illustrated in Fig. 1. If *w*^*t*^ is the first content word of the sentence, then it has no semantic similarity value as *A* is empty. This approach suffers from low mathematical or linguistic interpretability, as it is not clear what the summation of the vectors of the content words preceding a target word represents. Additionally, the exclusion of function words as context words may not be justified, as function words can affect the processing of the target word as well. Aside from the limitations of the cosine semantic similarity measure, the sample size of the study (i.e., the number of target words and stimuli sentences used) was relatively small, and the stimuli sentences were mostly presented in isolation without the surrounding discourse. As such, it would be desirable to validate the results pertaining to the predictability of the cosine semantic similarity measure for word processing using a larger number of target words and large-scale text stimuli representing naturalistic discourse.

**Fig. 2:**
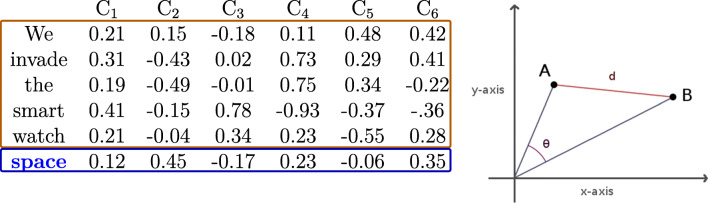
The Euclidean approach from Broderick et al. (2019). In the left table, the target word is “space”, and its preceding words are “we”, “invade”, “the”, “smart” and “watch”. Each word has its own word vector, and the word vectors of the preceding five words are summed and then averaged to yield a new vector, represented by “B” in the right panel. The vector of the target word “space” is represented by “A” in the right panel. “d” represents the Euclidean distance between A and B. The *Euclidean semantic similarity* value of the target word is calculated by dividing 1 by the Euclidean distance

Another approach for computing contextual semantic similarity based on word2vec embeddings used the *Euclidean distance* (Broderick, Anderson, Di Liberto, Crosse, & Lalor, 2018; Broderick et al., 2019). Specifically, the Euclidean semantic similarity value of a target word is computed by dividing 1 by the Euclidean distance between the vector of the target word and the averaged vector of all words preceding it in the sentence, as illustrated in Fig. 2. Several issues about this approach deserve further attention. First, the window size varies with the length of the sentence and the position of the target word in the sentence, making it somewhat problematic to compare the results of this approach with those from others with a fixed window size (e.g., three preceding words). Second, the approach also seems to lack mathematical and linguistic interpretability as it is unclear what the average vector derived from the word vectors of all preceding words represent. Third, Euclidean similarity seems not to work well for high-dimensional word vectors. The reason for this is that Euclidean similarity will increase as the number of dimensions of word vectors increases.

The applications of the semantic similarity methods in cognitive studies remain limited. For example, (Salicchi, Lenci, & Chersoni, 2021) measured the cosine semantic similarity between a target word *w* and its context *c*, which consists of its preceding words in the same sentence, and calculated the *Spearman correlation* between the cosine semantic similarity and the eye-tracking variables for *w* (including total reading time, first fixation duration, and number of fixations). Multiple pretrained databases of word embeddings were considered, including BERT, ELMo, fastText, GloVe, and GPT, and the eye-tracking variables were obtained from the Provo and GECO databases (Cop, Dirix, Drieghe, & Duyck, 2017; Luke & Christianson, 2018). Salicchi et al., (2021) reported very high correlations between cosine semantic similarity and the eye-tracking variables, especially with the BERT and GloVe models. Although these results suggest the usefulness of the cosine semantic similarity for understanding eye-movements in language comprehension tasks, there are several issues that deserve further attention. First, the method used to calculate the embedding of the context *c* was not explicitly specified (e.g., summation vs. averaging, and using all preceding words or a fixed number of preceding words). Second, given that the pre-trained word embeddings were trained on different datasets, it is unclear whether the comparisons reveal superiority of the training approach or training dataset, or both. Finally, as mentioned earlier, the correlation analysis is relatively weak for assessing the usefulness of semantic similarity for predicting eye-tracking variables.

In light of the insights from and limitations of existing approaches and studies discussed above, the current study sets out to achieve the following objectives. First, we propose a novel method for calculating contextual semantic similarity of target words in a way that is mathematically and linguistically interpretable, given the increasing focus on model interpretability in recent machine learning and computation research (Molnar, 2019; Biecek and Burzykowski, 2021; Søgaard, 2021; Thampi, 2022). Second, we evaluate the effectiveness of the semantic similarity measures generated using this approach for predicting eye-movements in reading obtained from a large-scale database with naturalistic discourse as the stimuli texts. Third, we employ advanced regression methods to compare the performance of our approach with that of cosine and Euclidean approaches, without concerning ourselves of comparing different word embedding models, as it has been done in many other studies.

## Materials and methods

### Materials

This study draws data from the Provo Corpus, a corpus of eye-tracking data with the accompanying predictability norms (Luke & Christianson, 2018). This corpus was chosen for a number of reasons. First, it uses whole passages as stimuli, which is better than using isolated sentences as whole passages better approximate naturalistic discourse. Specifically, 55 English short passages derived from a variety of sources were taken as the stimuli. Each passage contains an average of 50 words and 2.5 sentences, and the stimuli texts collectively contain 2689 word tokens and 1197 unique words. Second, the corpus recruited a large number of English native speakers (*n*= 84) to complete the reading tasks. Third, the corpus includes data on different types of eye-movement measures in reading. The present study employed two specific eye-movement metrics as response variables, namely, *first fixation duration*, which refers to the duration of only the first fixation on the target word, and *total fixation duration*, which refers to the summed duration of all the fixations on the target word. Finally, an eye-movement database serves the purposes of our study better than an EEG or fMRI database both because it adequately represents naturalistic discourse reading and because the simple and straightforward nature of the dataset lends itself better for the statistical models and methods used in the current study to assess and compare the performance of different measures of semantic similarity.

Considering the objectives of the current study and informed by the successful use of word2vec word embeddings in prediction tasks reported in previous studies (Frank & Willems, 2017; Broderick et al., 2019), we utilized a set of 1 million pretrained word2vec-style English word embeddings to train the *dynamic*, *cosine*, and *Euclidean* approaches to semantic similarity (see Section “Methods”). These word embeddings, available at https://fasttext.cc/docs/en/english-vectors.html, were pre-trained using fastText on Wikipedia 2017, the UMBC webbase corpus, and the statmt.org news dataset (16 billion tokens). We choose word2vec word embeddings (Mikolov, Grave, Bojanowski, Puhrsch, & Joulin, 2017) rather than BERT (or ELMo) word embedding databases for three reasons. First, word2vec word embeddings have been shown to perform stably in NLP and prediction in language processing and comprehension. Second, and more importantly, using BERT would make it difficult to determine whether any potential improvement in performance over cosine and Euclidean approaches comes from the dynamic approach proposed in our study or the BERT models.

### Methods

This study proposes a new dynamic approach to contextual semantic similarity and compares it to two existing approaches, namely, the *cosine* method from Frank & Willems (2017) and the *Euclidean* method from Broderick et al. (2019), all trained on the same pre-trained database of word embeddings. The *cosine* method proposed by Frank & Willems (2017), which considers content contextual words only, was detailed in Section “?? ??”. To facilitate more direct comparisons, we introduce a modified cosine approach, in which we consider both content and function contextual words. Given a target word, we add up the vectors of the three preceding words regardless of whether they are content or function words. We then use the same cosine approach as in Frank & Willems (2017) to process the two rows of vectors representing the target word and the sum of the vectors of the preceding three words. This value is taken as the *new cosine semantic similarity* value of the target word. As detailed in Section “?? ??”, the window size of context may vary greatly in the *Euclidean* approach proposed by Broderick et al. (2019). Again to facilitate more direct comparisons, we introduce a modified Euclidean approach, in which we only consider the three preceding words as the context of a target word. With this modification, the semantic similarity value computed using the approach from Broderick et al. (2019) is taken to be the *simpler Euclidean semantic similarity* value of the target word. It should be noted that these modifications to and local optimizations of the cosine and Euclidean approaches do not completely overcome the inherent weakness of the original approaches.

In the *dynamic* approach, the calculation of contextual semantic similarity is based on contextual words. We use the text stimuli from the Provo eye-tracking corpus to compute the contextual semantic similarity for an individual word. The vectors from the pretrained word embeddings representing the target word and its three preceding words form a matrix. We follow (Frank & Willems, 2017) to consider three preceding words to facilitate direct comparison with their cosine approach. The matrix is then transposed such that the columns represent the contextual words. A corresponding correlation matrix is then derived, in which each cell indicates the *Pearson correlation coefficient* between two neighboring words. Next, we use the diagonal to divide the correlation matrix into two triangular matrices. The upper and lower matrices are the mirror of each other and are equally representative of the correlation matrix. The present study thus uses the lower triangular matrix only. The summation of the values of all correlation coefficients in the lower triangular matrix is taken to be the semantic similarity value of the target word in the specific context, which distinguishes it from the other words. We also calculate a *simpler dynamic semantic similarity* measure, which is the sum of the values of the correlation coefficients between the target word and each of the three preceding words only.

**Fig. 3:**
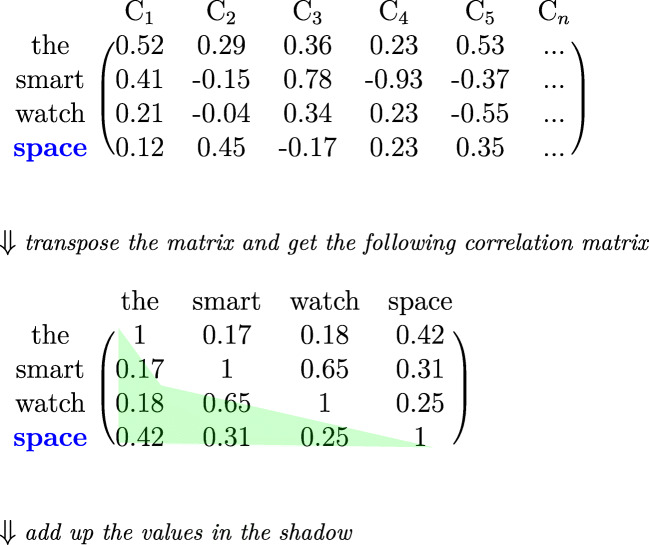
The *dynamic* approach for computing the semantic similarity of the target word “space”

Figure 3 illustrates the computation of the *dynamic semantic similarity* value of the target word “space”, which is preceded by “the”, “smart”, and “watch”. The four vectors representing the four words constitute a matrix shown in the top panel of the figure, with the value in each cell obtained from pretrained word embeddings. We transpose this matrix and then derive a corresponding correlation matrix, which is shown in the bottom panel of Fig. 3, where each cell represents the correlation coefficient between two neighboring words. For example, the correlation between “space” and “the” is 0.42. The values of the correlation coefficients in the lower triangular matrix under the diagonal (the green shadow in the figure) are then summed (i.e., 0.17 + 0.18 + 0.65 + 0.42 + 0.31 + 0.25 = 1.98). This value is taken to be the semantic similarity value of the target word in this context. Alternatively, to calculate the *simpler dynamic semantic similarity* measure, we add up the values of the correlation coefficients between “space” and each of the three preceding words only (i.e., 0.42 + 0.31 + 0.25 = 0.98).

The dynamic approach has two important advantages.^2^ First, as a novel attempt to assess the amount of “connectivity” between the target word and its neighboring words (including both function and content words), it generates semantic similarity measures that capture comprehensive contextual information in a mathematically and linguistically interpretable way. Second, this approach allows the semantic similarity values of a word to change dynamically in context. Overall, this study uses the three methods (i.e., *cosine*, *Euclidean*, and *dynamic*) to yield six types of contextual semantic similarity measures, as summarized in Table 1.

**Table 1:** The models and statistical analysis used in this study

Method	Measures	Words	Datasets	Statistical analysis
cosine	cosine semantic similarity (the method of (Frank & Willems, 2017))	content	dataset 1	GAMM, QGAM
cosine	new cosine similarity (same as (Frank & Willems, 2017) but considers both function and content words)	function & content	datasets 1 & 2	GAMM, QGAM
Euclidean	Euclidean semantic similarity (the method of Broderick et al. (2019)	function & content	datasets 1 & 2	GAMM, QGAM
Euclidean	simpler Euclidean similarity (same as Broderick et al. (2019) but considers the three preceding words only)	function & content	datasets 1 & 2	GAMM, QGAM
dynamic	dynamic semantic similarity (lower triangular matrix)	function & content	datasets 1 & 2	GAMM, QGAM
dynamic	simpler dynamic similarity (correlation row of the target word)	function & content	datasets 1 & 2	GAMM, QGAM

### Statistical methods and two datasets

We used *Generalized Additive Mixed Models* (GAMMs) (Wood, 2017) to address the first research question, namely whether the new semantic similarity measures can reliably predict the eye-tracking data. GAMMs are a type of mixed effects regression model. Compared to traditional regression methods, GAMMs are more effective for analyzing nonlinear effects and multiplicative interactions between variables making them especially appropriate for assessing the predictability of semantic similarity.

In addition to GAMMs, we used *Quantile GAM* (“qgam”) to address the second research question, namely, how the new semantic similarity measures compare with the measures computed by *cosine* and *Euclidean* approaches in predicting the eye-tracking data. We compared the *dynamic* approach to the cosine method and the Euclidean method. *Quantile regression* broadens the scope of regression analysis and makes it possible to study functional dependencies between the quantiles of a response and one or more predictors (Fasiolo, Wood, Zaffran, Nedellec, & Goude, 2020), taking into account the potential variation of the effect sizes of the predictors among the quantiles. The quantile regression was carried out using the “qgam” R package (Fasiolo et al., 2020).

When response variables are simple, these statistical methods are easier and more direct to carry out. Eye-tracking data has greater advantages than EEG and fMRI data in implementing these statistical methods. That is why eye-tracking data on naturalistic discourse reading was chosen as response variables in the present study. Model performance comparisons is complex, and correlation is difficult to assess model comparisons. By contrast, “qgam” definitely is well capable of assessing model performance comprehensively and precisely.

(Q)GAM model comparisons were implemented using the compareML function in the “itsadug” R package, a method that may be preferred over other functions such as AIC (Akaike information criterion) or BIC (Bayesian information criterion) for models that include random effects (Van Rij, Wieling, Baayen, & van Rijn, 2015). Nevertheless, we supplemented this method by comparing the AIC and BIC values of the models, using the criterion that a smaller AIC or BIC value indicates a better model.

The *cosine* semantic similarity measure only considered content words, while the other five measures considered both *content words* and *function words*. To facilitate a comprehensive comparison of the performance of the semantic similarity measures, we first built and compared models for all six measures on content words (dataset 1, 6549 tokens), the results of which are reported in Section “Results”; we then built and compared models for the five measures on both function and content words (dataset 2, 39210 tokens), the results of which are reported in the Supplementary Materials due to space constraints. Table 1 summarizes the six semantic similarity measures and the two datasets examined in the present study.

## Results

### Study 1: Predictability of dynamic semantic similarity

Using dataset 1, we fitted 12 GAMM models to analyze the six types of semantic similarity as predictors of two dependent variables (*first fixation duration* and *total fixation duration*). The main predictor of interest is modeled as a tensor product smooth. The models also include *word length* and *word frequency* as control predictors, modeled as tensor interaction, and *participant* as a random effect. The main predictor of interest is modeled as a tensor product smooth.

Table 2 summaries the 12 GAMM models fitted to the eye-tracking data. The semantic similarity data is largely normally distributed. With the exception of *Euclidean semantic similarity*, which only significantly predicts first fixation duration, the other five semantic similarity all significantly predict both first and total fixation duration. The tensor interaction of word length and word frequency also significantly predicts fixation duration, indicating that either variable is capable of predicting eye-movement in reading. The random effect of *participant* is significant in all GAMM models as well.

**Table 2:** Model summaries of the 12 GAMM models fitted to the eye-tracking data

Class	Parametric coefficients	Estimate	Std. Error	t-value	*p*-value
First fixation (dynamic)	intercept	213.96	3.18	67.34	< 2e-16 (***)
Total fixation (dynamic)	intercept	250.14	8	31.27	< 2e-16 (***)
First fixation (simpler dynamic)	intercept	213.61	3.39	63.09	< 2e-16 (***)
Total fixation (simpler dynamic)	intercept	250.95	7.94	31.6	< 2e-16 (***)
First fixation (cosine)	intercept	212.42	3.64	58.35	< 2e-16 (***)
Total fixation (cosine)	intercept	251.27	7.99	31.46	< 2e-16 (***)
First fixation (new cosine)	intercept	214.42	3.19	67.27	< 2e-16 (***)
Total fixation (new cosine)	intercept	251.73	7.92	31.8	< 2e-16 (***)
First fixation (Euclidean)	intercept	215.36	3.22	66.95	< 2e-16 (***)
Total fixation (Euclidean)	intercept	250.76	7.9	32.4	< 2e-16 (***)
First fixation (simpler Euclidean)	intercept	214.76	3.19	67.36	< 2e-16 (***)
Total fixation (simpler Euclidean)	intercept	258.17	7.58	34.04	< 2e-16 (***)
Class	Smooth Terms	edf	Ref.df	F-value	*p*-value
First fixation (dynamic semantic similarity)	te(word length, frequency)	3	3	7.3	< 2e-16 (***)
	s(dynamic semantic similarity)	6.96	8.06	17.43	< 2e-16 (***)
	s(participant, bs=“re”)	55.03	73	4.32	< 2e-16 (***)
Total fixation (dynamic semantic similarity)	te(word length, frequency)	4.38	4.92	129.56	< 2e-16 (***)
	s(dynamic semantic similarity)	7.9	8.69	9.85	< 2e-16 (***)
	s(participant, bs=“re”)	62.29	73	6.7	1.2e-05 (***)
First fixation (simpler dynamic similarity)	te(word length, frequency)	3.9	4.35	6.03	9.62e-05 (***)
	s(simpler dynamic similarity)	2.62	3.28	16.72	< 2e-16 (***)
	s(participant, bs=“re”)	55.02	73	4.39	< 2e-16 (***)
Total fixation (simpler dynamic similarity)	te(word length, frequency)	4.25	4.78	119.75	< 2e-16 (***)
	s(simpler dynamic similarity)	5.91	7.06	7.3	< 2e-16 (***)
	s(participant, bs=“re”)	62.26	73	6.61	1.4e-05(***)
First fixation (cosine semantic similarity)	te(word length, frequency)	4.5	5.06	4.65	0.0003(***)
	s(cosine semantic similarity)	6.57	7.7	6.23	< 2e-16 (***)
	s(participant, bs=“re”)	55.01	73	4.31	< 2e-16 (***)
Total fixation (cosine semantic similarity)	te(word length, frequency)	4.4	4.95	128.29	< 2e-16 (***)
	s(cosine semantic similarity)	1	1	33.19	< 2e-16 (***)
	s(participant, bs=“re”)	62.25	73	5.98	< 2e-16 (***)
First fixation (new cosine similarity)	te(word length, frequency)	3	3	7.51	5.23e-05(***)
	s(new cosine similarity)	4.7	5.83	6.02	5.44e-06 (***)
	s(participant, bs=“re”)	54.97	73	4.28	< 2e-16 (***)
Total fixation (new cosine similarity)	te(word length, frequency)	4.09	4.59	103.58	< 2e-16 (***)
	s(new cosine similarity)	5.39	6.6	3.62	0.001 (**)
	s(participant, bs=“re”)	62.29	73	6.54	1.64e-05 (***)
First fixation (Euclidean semantic similarity)	te(word length, frequency)	3	3	7.48	5.47e-05 (***)
	s(Euclidean semantic similarity)	6.13	7.25	2.88	0.0052 (*)
	s(participant, bs=“re”)	55.12	73	4.3	< 2e-16 (***)
Total fixation (Euclidean semantic similarity)	te(word length, frequency)	4.04	4.53	157.33	< 2e-16 (***)
	s(Euclidean semantic similarity)	2.62	3.29	2.21	0.075
	s(participant, bs=“re”)	62.33	73	6.51	1.074e-05 (***)
First fixation (simpler Euclidean similarity)	te(word length, frequency)	3	3	8.51	1.26e-05 (***)
	s(simpler Euclidean similarity)	3.29	4.15	6.51	2.43-05 (***)
	s(participant, bs=“re”)	54.98	73	4.28	< 2e-16 (***)
Total fixation (simpler Euclidean similarity)	te(word length, frequency)	3.39	3.63	178.16	< 2e-16 (***)
	s(simpler Euclidean similarity)	4.37	5.38	5.65	2.38e-05 (***)
	s(participant, bs=“re”)	62.38	73	6.54	1.68e-05 (***)

Two dependent variables (first fixation duration and total fixation duration) were predicted using six measures (six types of semantic similarity). te () = tensor interaction, s() = smoothing, re = random effect, n = 6548 (first fixation), *n* = 6548 (total fixation). * *p* <.01, ** *p* <.001, *** *p* <.0001)

**Fig. 4:**
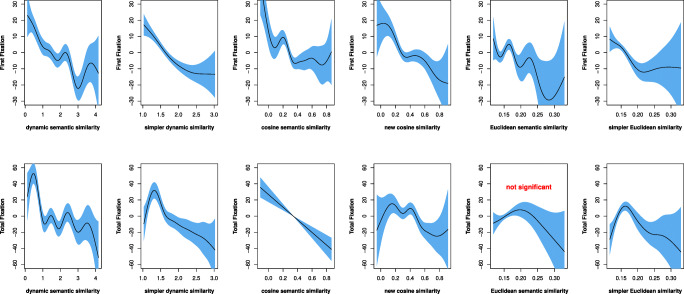
Fixed effects for contextual semantic similarity

Figure 4 presents the fixed effects of the semantic similarity variables on first fixation and total fixation in the GAMM models. It is clear that all significant fixed effects are negative. In other words, both first fixation duration and total fixation duration tend to decrease as semantic similarity increases. These results suggest that words that are less likely to occur in the same context have smaller contextual semantic similarity and require more time for language users to process, while those that are more likely to occur in the same context have larger semantic similarity and entail less time to process. Two additional observations can be made about these results. First, as semantic similarity grows larger, its effect on fixation duration tends to become less apparent. Second, there is some variation in the stages in which different types of semantic similarity take effect. For example, the *cosine semantic similarity* is activated earlier and more strongly than the other four significant predictors in the first fixation, but its effect quickly vanishes. Meanwhile, the two dynamic semantic similarity measures appear to have a more stable effect on first fixation than the other three significant predictors. These differences may have arisen because of the different approaches used to obtain semantic similarity information.

The results reported above have shown that *Euclidean semantic similarity* does not predict fixation duration as well as the other five types of semantic similarity. We further compare the performance of the models in two ways here: first using the compareML function in the “itsadug” R package (Van Rij et al., 2015) and second using BIC and AIC for models. These comparisons yield the same results, with the models with *dynamic semantic similarity* performing the best for both response variables.^3^ Specifically, for first fixation duration, the performance of the models is ranked as follows: *dynamic semantic similarity*> *simpler dynamic similarity*> *cosine semantic similarity*> *new cosine similarity*> *simpler Euclidean similarity* > *Euclidean semantic similarity*; for total fixation duration: *dynamic semantic similarity* > *simpler dynamic similarity*> *cosine similarity*> *simpler Euclidean similarity* > *new cosine similarity* > *Euclidean semantic similarity*.

The findings from the GAMM fittings in dataset 2, detailed in the Supplementary Materials section, similarly showed significant negative effects of semantic similarity on fixation duration, with the exceptions that *Euclidean semantic similarity* and *simpler Euclidean similarity* did not significantly predict first fixation duration. With the same two comparison methods described above, the performance of the models is ranked as follows for both response variables: *dynamic semantic similarity*> *simpler dynamic similarity*> *new cosine similarity*> *simpler Euclidean similarity* > *Euclidean semantic similarity*. These results corroborate those from dataset 1 that the two measures yielded by the *dynamic* approach are better predictors of eye-movements in reading than the measures yielded by the *cosine* and *Euclidean* approaches.

### Study 2: Comparison of the three approaches (six measures) in qgam models

**Fig. 5:**
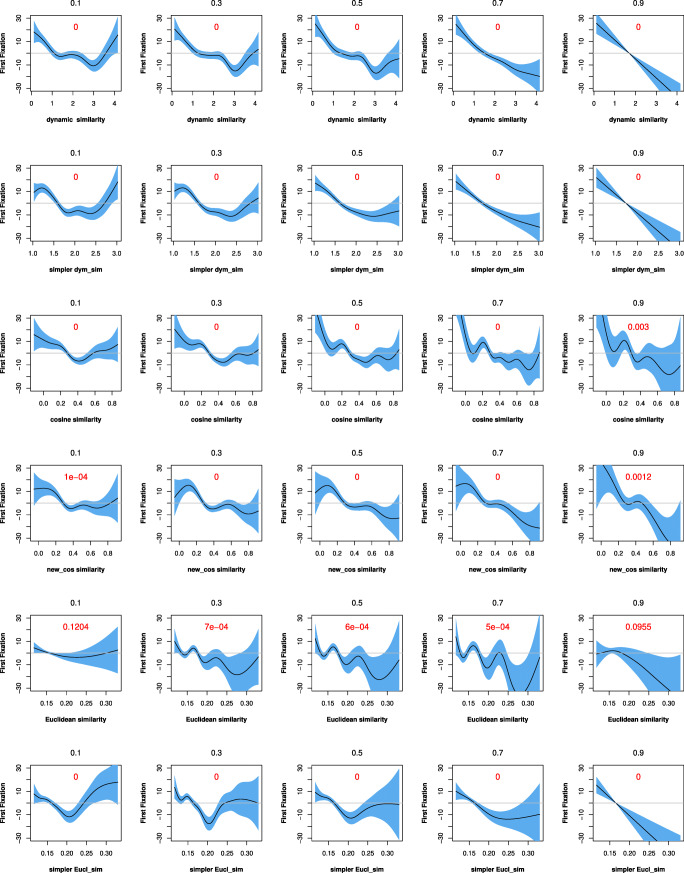
Fixed effects for semantic similarity in predicting first fixations with qgam models across deciles. Note: *simpler dyn_sim* = simpler dynamic similarity; *new_cos similarity* = new cosine similarity; *simpler Eucl_sim* = simpler Euclidean similarity

**Fig. 6:**
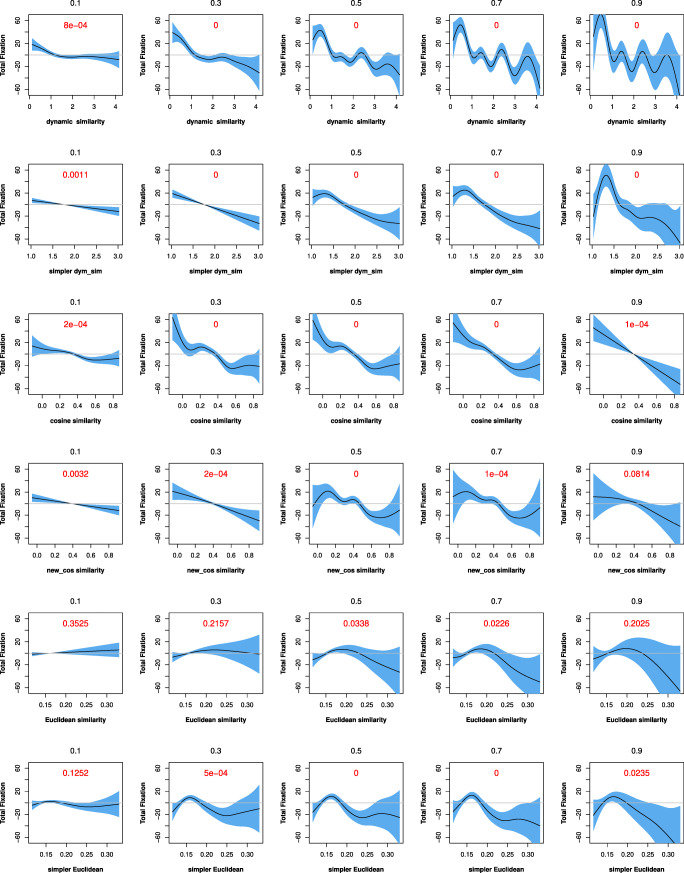
Fixed effects for semantic similarity in predicting the total fixations with qgam models across deciles. Note: *simpler dyn_sim* = simpler dynamic similarity; *new_cos similarity* = new cosine similarity; *simpler Eucl_sim* = simpler Euclidean similarity

In this section, we compared the performance of the six semantic similarity measures in qgam models, using the “qgam” R package to assess the effect sizes of the predictors, which vary with quantiles (0.1, 0.3, 0.5, 0.7, and 0.9). Two models were built for each semantic similarity measure on dataset 1, each with first fixation duration and total fixation duration as the response variable, respectively. Each model includes the main effect of semantic similarity, the tensor interaction of *word length* and *word frequency*, and the random effect of *participant*. The effects of each semantic similarity measure for predicting the two response variables across deciles are visualized in Figs. 5 and 6.

We first examined the *p* values and trends of fixed effect changes for the independent variables across deciles. In Figs. 5 and 6, a panel in each row represents how a given semantic similarity metric reacts at the five deciles. Models that vary between significant and insignificant effects across the five deciles are not as stable as those consistently show significant effects across the five deciles. We set the *alpha* value at .01 for this comparison. The results show that the effects of the two *dynamic* semantic similarity measures remain stable across deciles. The *cosine semantic similarity* measure is also stable, but the *new cosine similarity* measure has an insignificant effect at one decile. The two *Euclidean semantic similarity* measures are the least stable, with insignificant effects at multiple deciles. The changes of the effects for the metrics look similar, with some fluctuations at the beginning and the slope gradually becoming steep later, indicating strong effects. However, the curves of the *Euclidean semantic similarity* measure are likely to fluctuate at zero level, suggesting that the model does not converge well.

Next, we employed both compareML and BIC/AIC to make further model comparisons. We used compareML to compare models at the same decile. Although the performance of each model may vary across different deciles and/or for the two response variables, the overall performance of these models is very consistent. Specifically, for first fixation duration, the performance of the models is ranked as follows: *dynamic semantic similarity*> *simpler dynamic similarity*> *simpler Euclidean similarity* > *new cosine similarity* > *cosine semantic similarity* > *Euclidean semantic similarity*. For total fixation duration, the performance of the models is ranked as follows: *dynamic semantic similarity*> *simpler dynamic similarity*> *cosine semantic similarity*> *new cosine similarity*> *simpler Euclidean similarity* > *Euclidean semantic similarity*.

We also calculated the BIC and AIC values for these models at the same decile. With the criterion that smaller values of BIC or AIC correspond to better performance, the performance of these models is ranked consistently for all deciles and for both response variables, as follows: *dynamic semantic similarity*> *simpler dynamic similarity*> *cosine semantic similarity*> *new cosine similarity*> *simpler Euclidean similarity* > *Euclidean semantic similarity*.

Overall, the performance of the semantic similarity metrics in the qgam models is largely consistent with the results of model comparisons using compareML and BIC/AIC, pointing to the same overall ranking of the models’ performance as reported in Section “?? ??”: *dynamic semantic similarity*> *simpler dynamic similarity*> *cosine semantic similarity*> *new cosine similarity*> *Euclidean semantic similarity* > *simpler Euclidean similarity*.

The fixed effects of the six semantic similarity measures in the qgam models on dataset 2, detailed in the Supplementary Materials Section, are consistent with those on dataset 1. In particular, with the same comparison methods described above, we can obtain the same ranking of the models’ performance as in dataset 1, confirming the results from dataset 1 that the two *dynamic* semantic similarity measures are better predictors of eye-movements in reading than the measures yielded by the *cosine* and *Euclidean* approaches.

## Discussion

To summarize, our analyses of dataset 1, which considers content words as contextual words, and dataset 2, which considers both function and content words as contextual words, using GAMM and QGAM models yielded fairly consistent results. The two contextual semantic similarity measures computed using our novel, dynamic approach performed the best in predicting both first fixation durations and total fixation durations. The two *cosine similarity* measures were both predictive of both response variables as well. The *simpler Euclidean similarity* measure performed better than the *Euclidean semantic similarity* measure: the former did not significantly predict one response variable in dataset 2, while the latter did not significantly predict one response variable in both datasets.

A major contribution of the current study is methodological. Our novel approach to contextual semantic similarity not only outperforms existing cosine and Euclidean approaches (Frank & Willems, 2017; Broderick et al., 2019) but also is both mathematically and linguistically interpretable. In particular, among the six measures compared, the *dynamic semantic similarity* measure is the most predictive of first and total fixation duration as well as the most stable across deciles. The main reason may be that each cell in the triangular matrix meaningfully represents the correlation between the target word and a contextual word, and the sum of those correlations thus meaningfully represent the overall degree of semantic similarity between the target word and the contextual words within the context window. By contrast, the summation or average of the word embeddings of the preceding words are more difficult to interpret mathematically or linguistically. A second reason may be the consideration of both content and function words as contextual words, as supported by the better results of the new cosine similarity measure we proposed, which also uses both types of contextual words, than the original cosine similarity measure, which considers content words only. A third reason may be the use of a fixed window size for the context (i.e., three preceding words), as supported by the better results of the *simpler Euclidean similarity* measure we proposed, which uses a window size of three, than the original *Euclidean semantic similarity* measure, which considers all words preceding the target word in the sentence.

Overall, our findings confirm the predictive effects of contextual semantic similarity on eye-movements in reading, as have reported in previous studies of the relationship between semantic similarity and eye-movements (or reading/comprehension difficulty) (e.g., Roland et al., 2012; Broderick et al., 2019). More specifically, target words with greater semantic similarity to their contextual words are processed faster. This finding can be explained by how contextual semantic similarity based on word embeddings captures the syntagmatic dimension of the semantic relationship between a target word and its surrounding words within a sentence instead of the paradigmatic dimension of the semantic relationship between a target word and other words of the same category that could replace it in the same context as claimed by Frank & Willems (2017). For example, the words “man”, “play”, and “ball” are syntagmatically related, and they need to be arranged in a particular order in English, whereas the words “ball” and “golf” are paradigmatically related, as they could replace each other in many contexts. Knowledge of the syntagmatic structure allows the language user to predict that “the ball” may follow “the man will play”. It is by modeling syntagmatic relations that the approaches to contextual semantic similarity discussed in the current study are capable of making probabilistic word predictions, similar to how the *surprisal* approaches can calculate word probabilities on the syntagmatic level (Hale, 2001; Demberg & Keller, 2008; Smith & Levy, 2013), as corroborated by Sun and Nixon (2022)’s finding that the *surprisal* measure and semantic similarity measure interact with each other in predicting words processing in context. As greater contextual semantic similarity leads to higher probability of the target word in the context, it is not surprising that it also leads to shorter fixation duration or reading time in general.

Our comparison of the six types of contextual semantic similarity metrics suggested that while the effect of contextual semantic similarity may take place in both early and late stages of language processing, not all metrics may capture this effect in both stages, as shown in Figs. 4 and 7. Some of our findings in this regard align with those reported in previous studies. For example, Yan and Jaeger (2020) used the cosine approach (Frank & Willems, 2017) to compute semantic similarity and reported that contextual information affects ERPs in both early (200ms after word onset) and late (N400) time windows. Our results pertaining to *cosine semantic similarity* (see Fig. 4) are largely consistent with their finding. Broderick et al., (2019) found that the early cortical tracking of a word’s speech envelope is enhanced by its semantic similarity to its sentential context, and this effect manifested in the prediction accuracy of the EEG signals. We found similar results for Broderick et al., (2019)’s *Euclidean semantic similarity* measure in predicting eye-movements in reading. In short, though, for all six contextual semantic similarity measures, the effect of semantic similarity on fixation duration tends to be stronger in the initial stage of processing and generally becomes less apparent as it continues to become larger (see Fig. 4), similar to the findings reported by Roland et al., (2012).

## Conclusions

This study constitutes the first endeavor to compare the performance of multiple approaches to contextual semantic similarity in predicting eye-tracking variables in language comprehension and processing. We proposed an interpretable approach for computing contextual semantic similarity based on word embeddings. Using a database of eye-tracking data with naturalistic discourse as text stimuli and additive mixed-effect regression models, we confirmed that the contextual semantic similarity measures computed by our novel, dynamic approach significantly predict eye-movements in reading. We further showed that the measures computed by our approach outperform those computed by the *cosine* and *Euclidean* approaches (Frank and Willems, 2017; Broderick et al., 2018). Although we focused on eye-tracking data in English in the current study, the approach proposed in this study can be applied in future research to explore eye-tracking data in other languages as well as data from EEG or fMRI experiments on language processing and comprehension.^4^ Such explorations will validate and usefully expand our findings to achieve a more comprehensive understanding of the relationship of contextual semantic similarity to language comprehension and processing. Future research can also examine how contextual semantic similarity interacts with other prediction models (e.g., *surprisal* measures) in predicting language comprehension and processing.

## Electronic supplementary material

Below is the link to the electronic supplementary material.

(PDF 357 KB)

## Footnotes

1 The number of preceding words considered was *three* in their method (Dr. Frank, personal communication).

2 The code for implementing the approach of dynamic semantic similarity and a data sample are available at: https://osf.io/3xksg/.

3 Detailed results of all compareML and BIC/AIC analyses in this study can be found at https://osf.io/zpjxu.

4 For example, we used these semantic similarity metrics to predict the newly released large-scale eye-movement data on reading Chinese sentences (Zhang, Yao, & et al. 2022), and achieved similar results to English. Moreover, after the same semantic metrics having been applied in predicting the eye-movement data on reading in 13 languages (Siegelman et al., 2022), we received the same results. The dynamic semantic similarity far outperfroms the surprisal measure in predicting the eye-movement data in these languages. We will report the results in another paper.
