# Supplementary material for: An interpretable measure of semantic similarity for predicting eye movements in reading
Source: Psychon Bull Rev. 2023 Feb 2;30(4):1227–42. doi: 10.3758/s13423-022-02240-8 (PMC10482772; doi:10.3758/s13423-022-02240-8)
Supplement: Supplementary file 1 — (PDF 357 KB) [file 13423_2022_2240_MOESM1_ESM.pdf]

Supplementary Materials

We explored `dataset 2` using content and function words. In this dataset, the *consine semantic similarity* adopted from Frank and Willems (2017) is excluded. We employed five measures: *dynamic semantic similarity*, *simpler dynamic similarity*, *new cosine similarity*, *Euclidean semantic similarity* and *simpler Euclidean similarity*. The statistical methods are the same as those used in the main text.

A The predictability of dynamic semantic similarity from dataset 2

We fitted 10 GAMM models to analyze the five types of semantic similarity as predictors of two dependent variables (first fixation duration and total fixation duration). The main predictor of interest is modeled as a tensor product smooth. The models also include word frequency length and word frequency as control predictors, modeled as tensor interaction, and participant as a random effect. Note that the size of `dataset 2` (39210 tokens) is much larger than that of `dataset 1` (6548 tokens).

Table 3 summaries the 10 GAMM models fitted to the eye-tracking data. The semantic similarity data is largely normally distributed. With the exceptions of *Euclidean semantic similarity* and *simpler Euclidean similarity*, which only significantly predict first fixation duration, the other semantic similarity measures all significantly predict both first and total fixation duration. The tensor interaction of word length and word frequency also significantly predicts fixation duration, indicating that either variable is capable of predicting eye-movement in reading. The random effect of *participant* is significant in all GAMM models as well.

Figure 7 presents the fixed effects of the semantic similarity variables on first fixation and total fixation in the GAMM models. As is the case for `dataset 1`, all significant fixed effects are negative. In other words, both first fixation duration and total fixation duration tend to decrease as semantic similarity increases. These results suggest that words that are less likely to occur in the same context have smaller contextual semantic similarity and require more time for language users to process, while those that are more likely to occur in the same context have larger semantic similarity and entail less time to process. When content and function words are both examined here, the fixed effects of both dynamic semantic similarity measures appear stronger than those on `dataset 1` (see Figure 4).

Using `compareML` and `BIC/AIC` and referring to the fixed effect of semantic similarity metric, we obtained the following ranking of performance of the GAMM models: *dynamic semantic similarity* > *simpler dynamic similarity* > *new cosine*

For Review Only

Table 3: Model summaries of the ten GAMM models fitted to the eye-tracking data. (Note: Two dependent variables (first fixation duration and total fixation duration) were predicted using the five measures (five types of semantic similarity). `te()` = tensor interaction, `re()` = random effect, `s()` = smoothing, n = 39210 (first fixation), n = 39210 (total fixation). * p < .01, ** p < .001, *** p < .0001.)

Class	Parametric Coefficients	estimate	Std.Erro	t-value	*p*-value
First fixation (dynamic)	intercept	208.12	2.82	73.88	<2e-16 (***)
Total fixation (dynamic)	intercept	196.93	9.63	20.45	<2e-16 (***)
First fixation (simpler dynamic)	intercept	209.73	2.7	77.63	< 2e-16 (***)
Total fixation (simpler dynamic)	intercept	202.62	11.04	18.36	< 2e-16 (***)
First fixation (new cosine)	intercept	207.65	3.36	61.72	< 2e-16 (***)
Total fixation (new cosine)	intercept	201.51	9.32	21.63	< 2e-16 (***)
First fixation (Euclidean)	intercept	207.07	3.4	61	< 2e-16 (***)
Total fixation (Euclidean)	intercept	194.79	8.98	21.7	< 2e-16 (***)
First fixation (simpler Euclidean)	intercept	208.45	2.85	73.2	< 2e-16 (***)
Total fixation (simpler Euclidean)	intercept	195.98	10.42	18.81	< 2e-16 (***)

Class	Smooth Terms	edf	Ref.df	F-value	*p*-value
First fixation (dynamic semantic similarity)	te(word length, frequency)	5.1	5.71	27	< 2e-16 (***)
	s(dynamic semantic similarity)	3.15	3.97	25.59	< 2e-16 (***)
	s(participant, bs="re")	70.15	73	43.79	< 2e-16 (***)
Total fixation (dynamic semantic similarity)	te(word length, frequency)	11.45	12.8	511.86	< 2e-16 (***)
	s(dynamic semantic similarity)	8.24	8.84	21.95	< 2e-16 (***)
	s(participant, bs="re")	71.27	73	242.72	< 2e-16 (***)
First fixation (simpler dynamic similarity)	te(word length, frequency)	3.87	4.1	38.64	<2e-16 (***)
	s(simpler dynamic similarity)	2.83	3.56	13.34	< 2e-16 (***)
	s(participant, bs="re")	70.14	73	43.38	< 2e-16 (***)
Total fixation (simpler dynamic similarity)	te(word length, frequency)	12.34	13.9	478.81	<2e-16 (***)
	s(simpler dynamic similarity)	8	8.73	17.09	< 2e-16 (***)
	s(participant, bs="re")	71.27	73	244.35	< 2e-16 (***)
First fixation (new cosine similarity)	te(word length, frequency)	6.65	7.13.63	20.5	< 2e-16 (***)
	s(new cosine similarity)	3.28	4.17	7.7	2.64e-6 (***)
	s(participant, bs="re")	70.14	73	43.07	< 2e-16 (***)
Total fixation (new cosine similarity)	te(word length, frequency)	11.02	12.37	475.22	< 2e-16 (***)
	s(new cosine similarity)	3.94	4.96	17.27	< 2e-16 (***)
	s(participant, bs="re")	71.27	73	240.04	< 2e-16 (***)
First fixation (Euclidean semantic similarity)	te(word length, frequency)	6.61	7	27.63	< 2e-16 (***)
	s(Euclidean semantic similarity)	1.61	2.03	0.53	0.58 (not significant)
	s(participant, bs="re")	70.12	73	42.98	<2e-16 (***)
Total fixation (Euclidean semantic similarity)	te(word length, frequency)	10.71	12.05	580.77	< 2e-16 (***)
	s(Euclidean semantic similarity)	2.68	3.39	3.73	0.0084 (**)
	s(participant, bs="re")	71.26	73	238.69	< 2e-16 (***)
First fixation (simpler Euclidean similarity)	te(word length, frequency)	5.48	6.29	29.17	< 2e-16 (***)
	s(simpler Euclidean similarity)	2.63	3.34	2.86	0.036
	s(participant, bs="re")	70.13	73	43.05	< 2e-16 (***)
Total fixation (simpler Euclidean similarity)	te(word length, frequency)	11.77	13.29	510.86	<2e-16 (***)
	s(simpler Euclidean similarity)	7.18	8.22	7.43	< 2e-16 (***)
	s(participant, bs="re")	71.27	73	240.45	< 2e-16 (***)

For Review Only

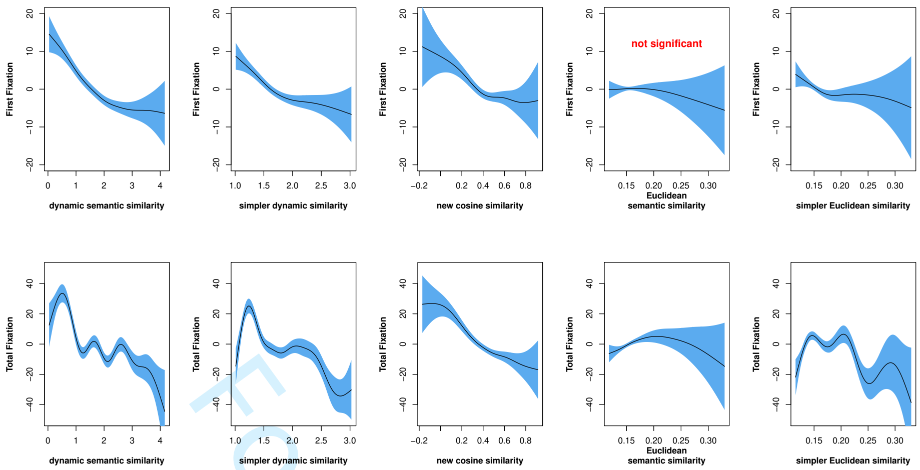

Figure 7: Fixed effects for contextual semantic similarity.

similarity > simpler Euclidean similarity> Euclidean semantic similarity. This ranking is consistent with that on `dataset 1`.

B Comparison of the five measures in qgam models

In this section, we compared the performance of the five semantic similarity measures in `qgam` models on `dataset 2`. Two models were built for each semantic similarity measure, each with first fixation duration and total fixation duration as the response variable, respectively. Each model includes the main effect of semantic similarity, the tensor interaction of *word length* and *word frequency*, and the random effect of *participant*. The effects of each semantic similarity measure for predicting the two response variables across deciles are visualized in Figures 8 and 9.

We first examined the p-values and trends of fixed effect changes for the independent variables across deciles. In Figures 8 and 9, a panel in each row represents how a given semantic similarity metric reacts at the five deciles. Models that vary between significant and insignificant effects across the five deciles are not as stable as those that consistently show significant effects across the five deciles. We set the *alpha* value at .01 for this comparison. The results show that the effects of the two *dynamic* semantic similarity measures and the two *cosine* similarity measures remain stable across deciles. In contrast, the *simpler Euclidean similarity* measure has an insignificant effect at one decile, while the *Euclidean*

For Review Only

similarity measure has an insignificant effect at nine deciles. The changes of the effects for the metrics look similar, with some fluctuations at the beginning and the slope gradually becoming steep later, indicating strong effects. However, the curves of the Euclidean semantic similarity measure are likely to fluctuate at zero level, suggesting that the model does not converge well.

Next, we employed both `compareML` and `BIC/AIC` to make further model comparisons. Both `compareML` and `BIC/AIC` were taken to compare models at the same decile. Although the performance of each model may vary across different deciles and/or for the two response variables, the overall performance of these models is very consistent. Specifically, for both first and total fixation duration, the performance of the models is ranked as follows: *dynamic semantic similarity* > *simpler dynamic similarity* > *new cosine similarity* > *simpler Euclidean similarity* > *Euclidean semantic similarity*. We also calculated the `BIC` and `AIC` values for these models at the same decile. With the criterion that smaller values of `BIC` or `AIC` correspond to better performance, the performance of these models is ranked consistently for all deciles and for both response variables, as follows: *dynamic semantic similarity* > *simpler dynamic similarity* > *simpler Euclidean similarity* > *new cosine similarity* > *Euclidean semantic similarity*.

Overall, the performance of the semantic similarity metrics in the `qgam` models is largely consistent with the results of model comparisons using `compareML` and `BIC/AIC`, pointing to the same overall ranking of the performance of the GAMM models on `dataset 2`: *dynamic semantic similarity* > *simpler dynamic similarity* > *new cosine similarity* > *simpler Euclidean similarity* > *Euclidean semantic similarity*.

For Review Only

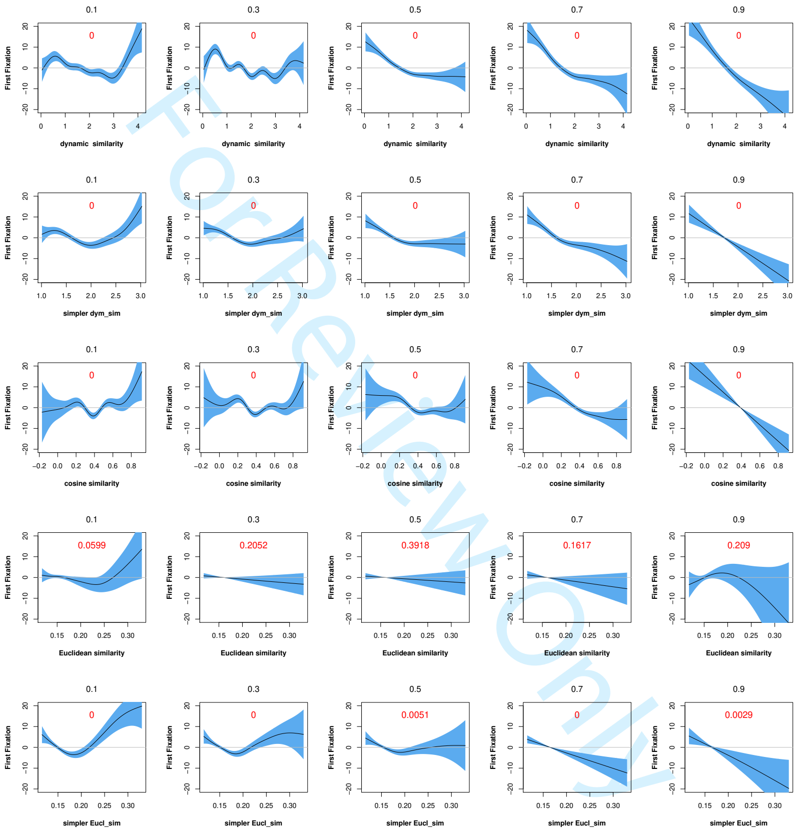

Figure 8: Fixed effects for semantic similarity in predicting the first fixations with qgam models. Note: *simpler dyn_sim* = simpler dymanic similarity; *cosine similarity* = new cosine similarity; *simpler Eucl_sim* = simpler Euclidean similarity.

For Review Only

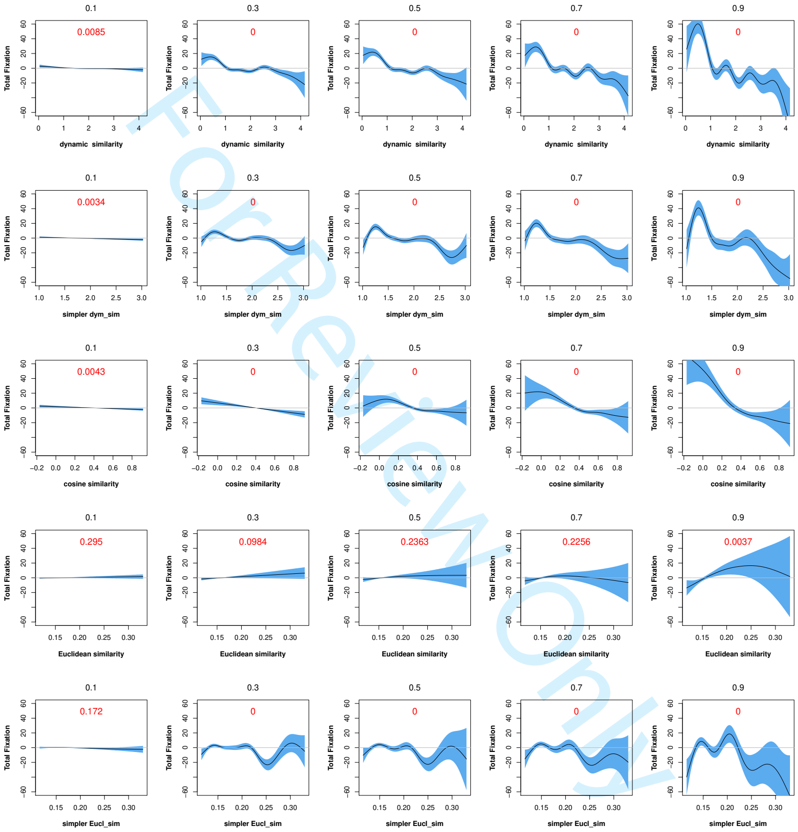

Figure 9: Fixed effects for semantic similarity in predicting the total fixations with qgam models. Note: *simpler dyn_sim* = simpler dynamic similarity; *cosine similarity* = new cosine similarity; *simpler Eucl_sim* = simpler Euclidean similarity.

For Review Only
